# Supplementary material for: Oxidized Mitochondrial DNA Engages TLR9 to Activate the NLRP3 Inflammasome in Myelodysplastic Syndromes
Source: Int J Mol Sci. 2023 Feb 15;24(4):3896. doi: 10.3390/ijms24043896 (PMC9966808; doi:10.3390/ijms24043896)
Supplement: Supplementary file 1 [file ijms-24-03896-s001.zip › ijms-2112604-supplementary.pdf]

Supplemental Figure S1: Oxidized mitochondria DNA Synthesis.

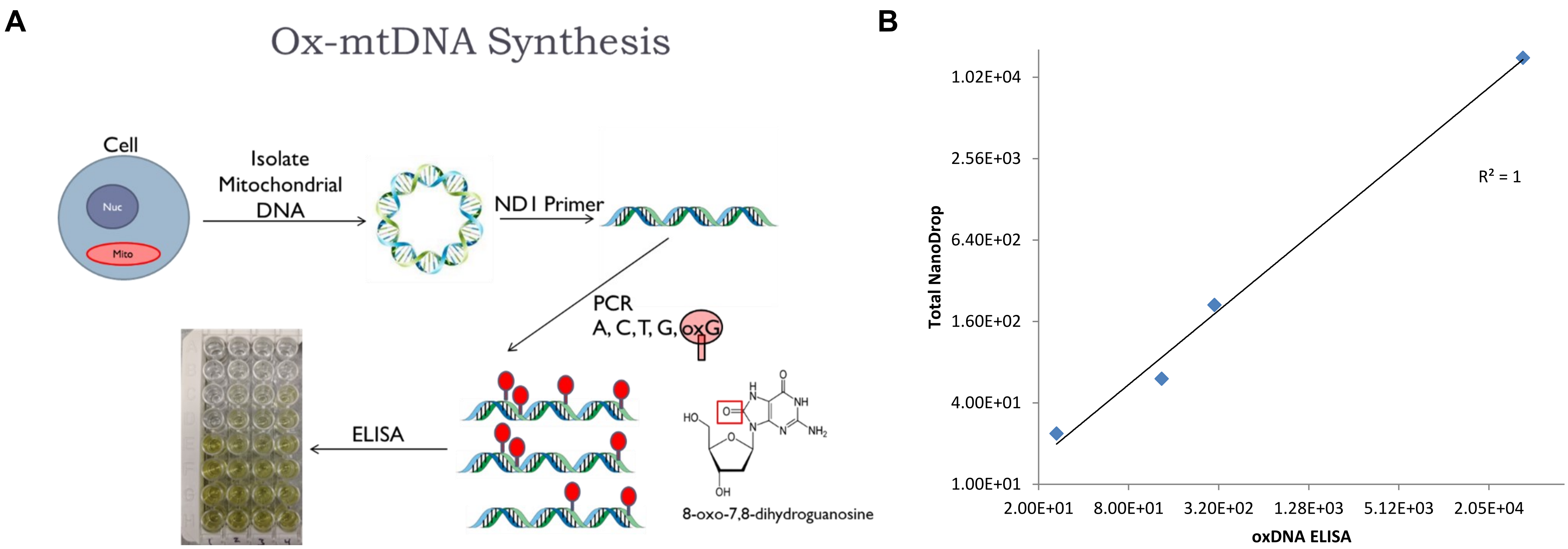

A) Schematic: Mitochondrial DNA was isolated from THP-1 cells via the Mitochondrial Extraction Kit according to the manufacture's protocol (Active Motif, Carlsbad CA) and amplified by the mitochondrial specific ND1 primers (ND1 Forward: 5′-CCCTAAAACCCGCCACATCT-3′; ND1 Reverse: 5′-GAGCGATGGTGAGAGCTAAGGT-3′) with the addition of oxidized guanosine to the nucleotide master mix. **B) Quantification:** Synthesized ox-mtDNA was added to the DNA/RNA Oxidative Damage (High Sensitivity) ELISA Kit (Cayman Chemical Company, Ann Arbor, MI) to confirm oxidation.

Supplemental Figure S2: Oxidized mitochondria DNA activates IL-1β irrespective of dose

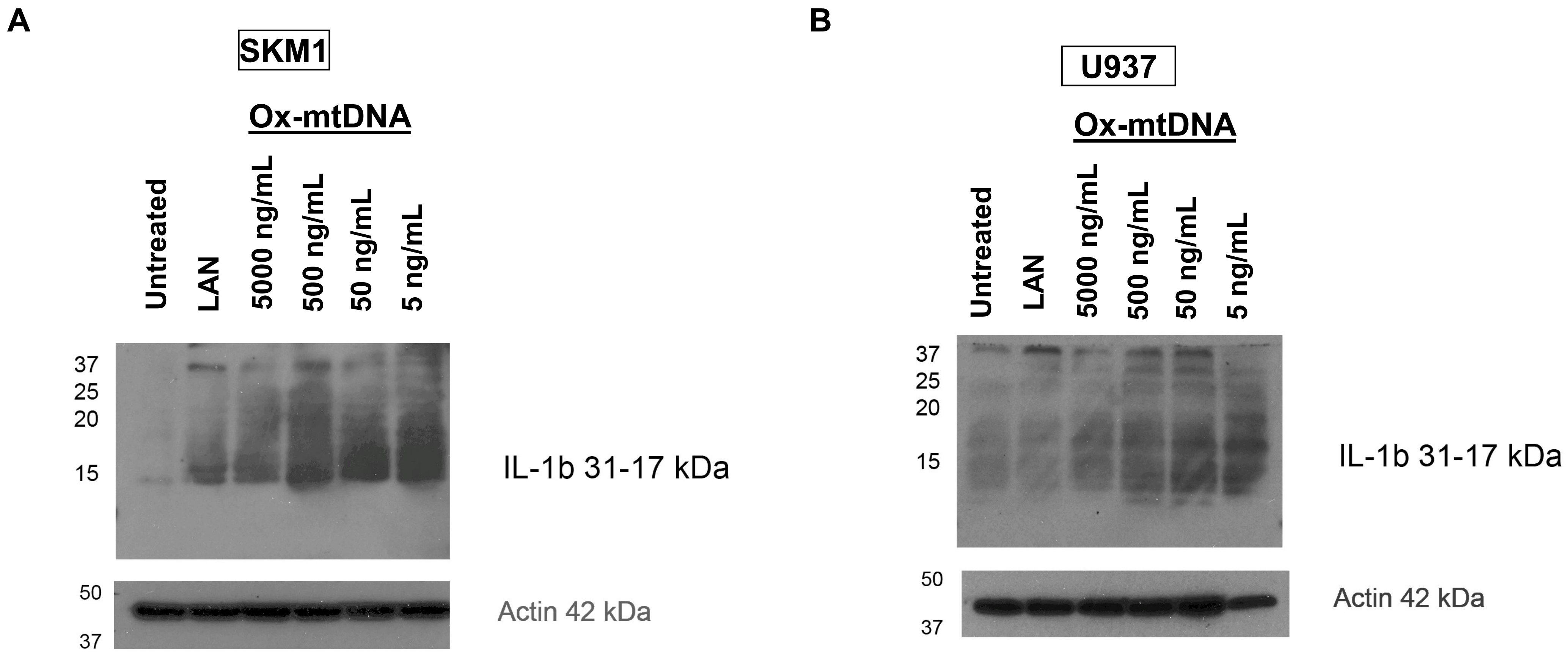

Experiment as in Figure 1B testing different doses of ox-mtDNA (as shown) on SKM1 **(A)** or U937 cells **(B)** followed by assessment of IL-1β activation (cleavage at 17kDa). LAN (LPS + ATP + Nigericin) was used as a positive control. Figures are representative of n=3 experimental repeats.

Supplemental Figure S3: Oxidized mitochondria DNA is dependent on the NLRP3 inflammasome

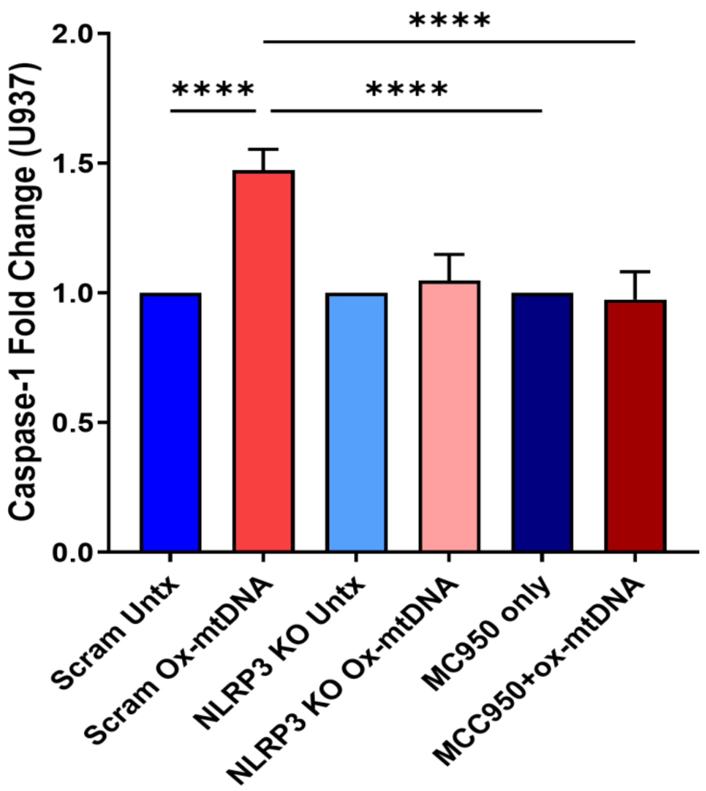

Repeat of experiment as in Figure 1F in U937 cells transfected with a lentivirus vector containing a pool of NLRP3 CRISPR KO guides, or a scrambled control or treated with 10uM of the NLRP3 specific inhibitor MCC950 for 24 hours prior to treatment with ox-mtDNA. Significance was assessed by ordinary one-way ANOVA with multiple comparison analysis in Graphpad Prism. P values are shown as asterisk: * $P \leq 0.05$, ** $P \leq 0.01$, *** $P \leq 0.001$, **** $P \leq 0.0001$.

Supplemental Table S1: qPCR primer sequences

Primer name	Sequence
ISG15 Forward	TCCTGCTGGTGGTGGACAAATG
ISG15 Reverse	CCGCTCACTTGCTGCTTCAGGT
CXCL10 Forward	TTGTCCACGTGTTGAGATCATTGCTAC
CXCL10 Reverse	AGACCTTTCCTTGCTAACTGCTTTCA
IFNα1 Forward	GATCTTCAACCTCTTTACCACAA
IFNα1 Reverse	ACACAGGCTTCCAAGTCATTCA
IFNα10 Forward	AGGGCCTTGATACTCCTGGGACAAAT
IFNα10 Reverse	TGGCTTGAGCCTTCTGGAACTGGT
IFNβ1 Forward	AGAAGGAGGACGCCGCATTGAC
IFNβ1 Reverse	TGATAGACATTAGCCAGGAGGTTC
TLR9 Forward	TGAGCCACAACTGCATCTCGCA
TLR9 Reverse	CAGTCGTGGTAGCTCCGTGAAT
ISG15 Forward	GAGAGGCAGCGAACTCATCT
ISG15 Reverse	CTTCAGCTCTGACACCGACA
CXCL10 Forward	AAGTGGCATTCAAGGAGTACCT
CXCL10 Reverse	AACACGTGGACAAAATTGGCT
SAMD9L Forward	AGGAAAATCCTGCATTTCCAGAG
SAMD9L Reverse	GCAAGGGGCTTACACTTTCC
CCL5 Forward	CCCTCACCATCATCCTCACT
CCL5 Reverse	AGAGGTAGGCAAAGCAGCAG
GAPDH Forward	GAAGGTGAAGGTCGGACT
GAPDH Reverse	GAAGATGGTGATGGGATTTC

Supplemental Figure S4: Ox-mtDNA and TLR9 are increased and colocalize in MDS

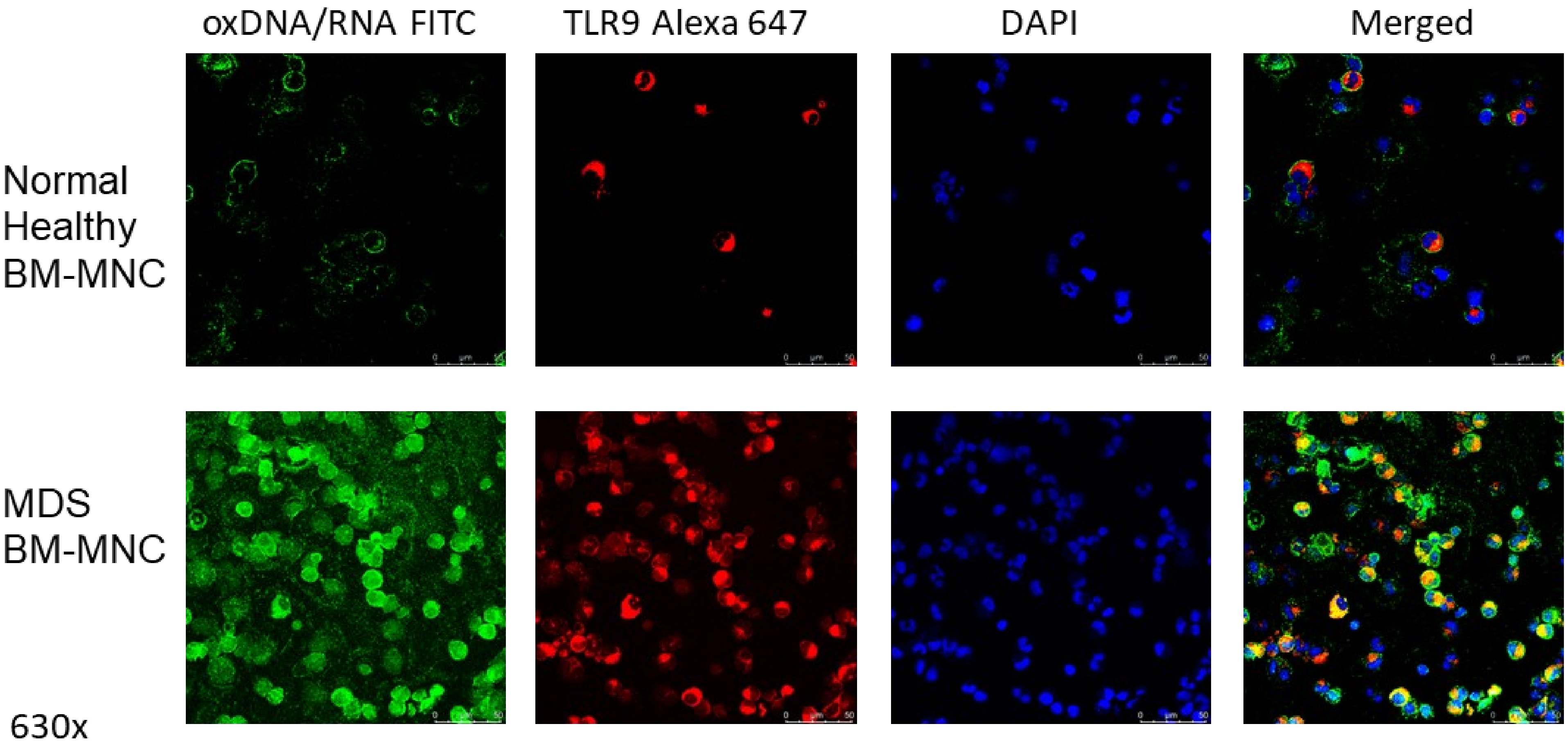

Lower magnification (630x) of the same data as in Figure 2A showcasing single colors and merged.

Supplemental Figure S5: Gating strategy for TLR9 expression in live primary HSPC

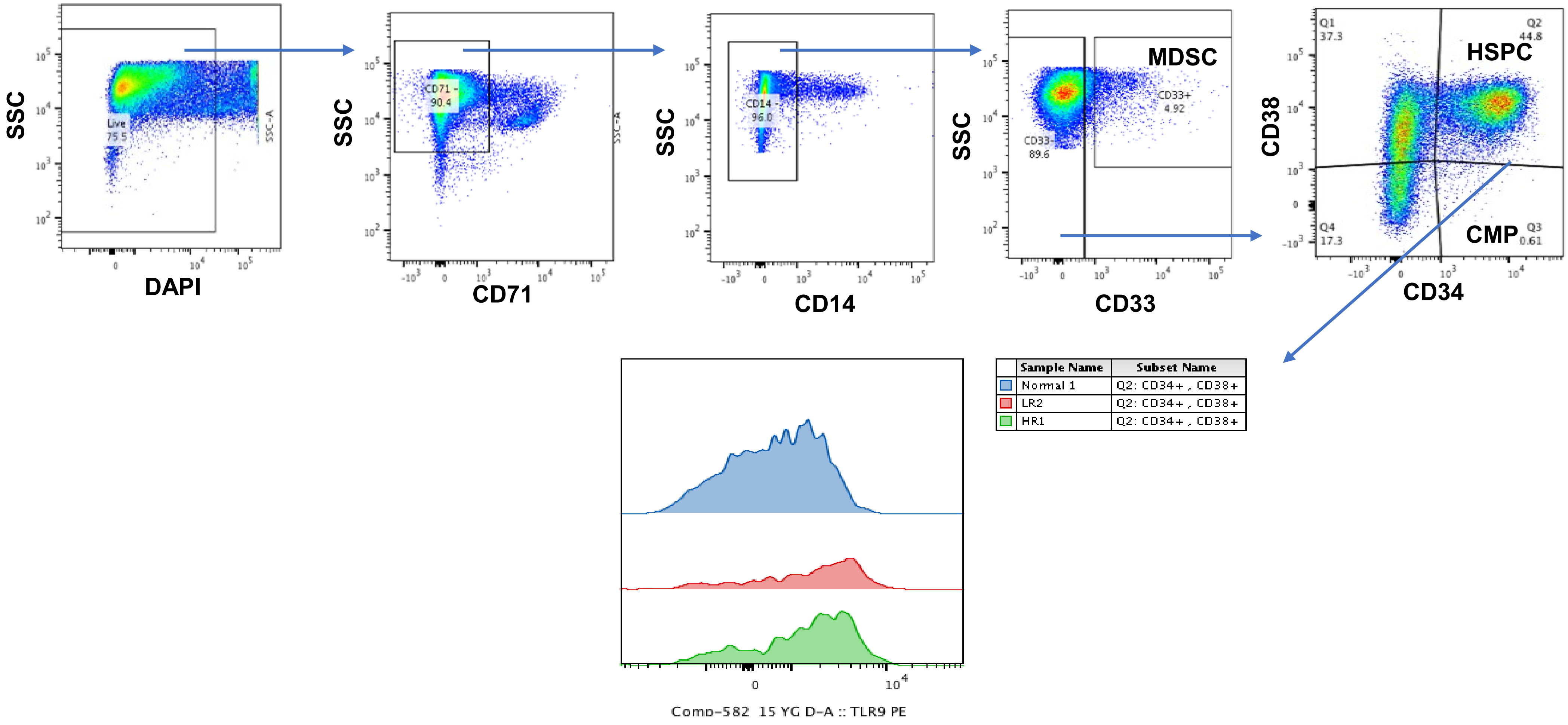

Gating strategy used to quantify the percent of cells with TLR9 surface expression and their subpopulations. HSPC are defined as Lineage-CD34+CD38+ cells.

Supplemental Figure S6: Gating strategy for TLR9 expression in live primary MDS specimens tSNE analysis

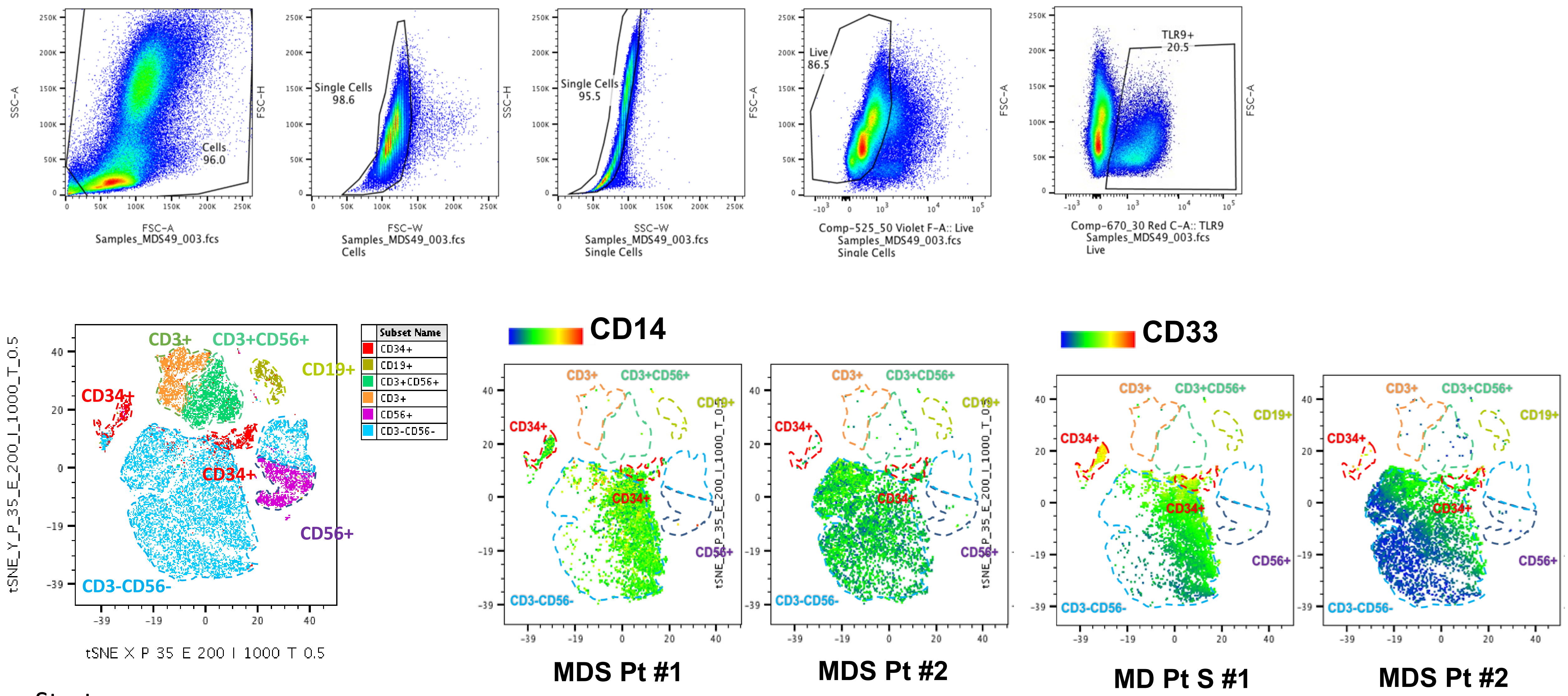

- Strategy:

1) Concatenate samples needed to be compared; 2) Identification of subpopulation by manual gating; 3) Apply to individual sample

Supplemental Figure S7: Ox-mtDNA and TLR9 are increased and colocalize in MDS

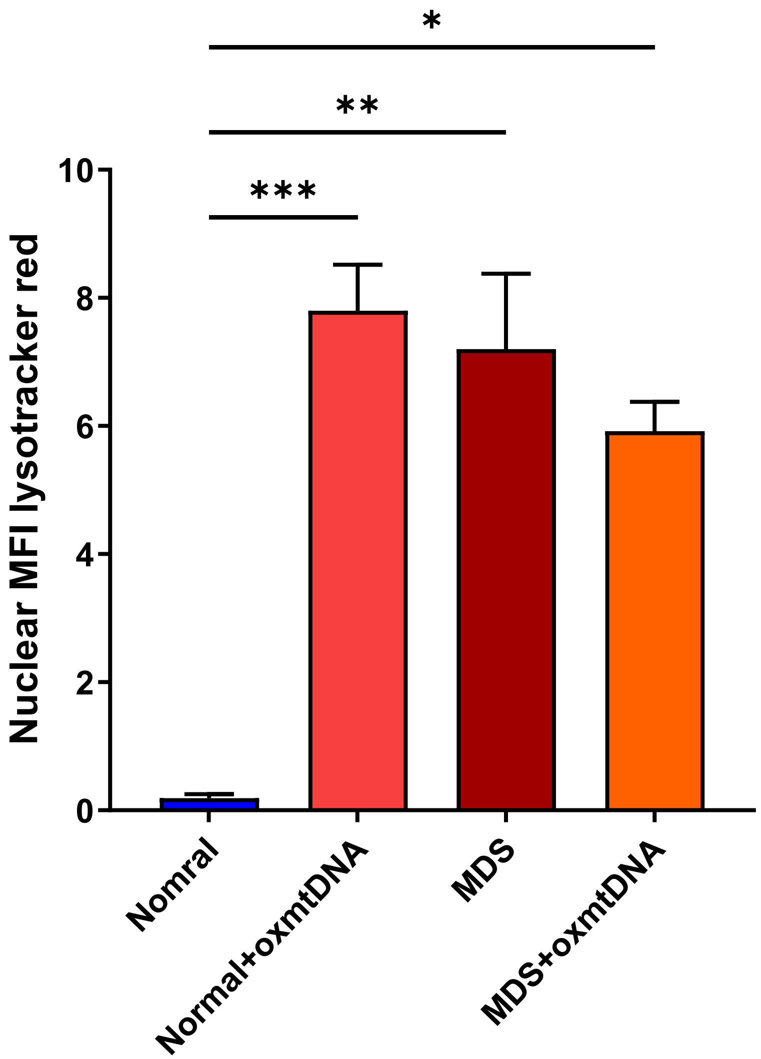

Quantification nuclear lysotracker MFI in the experiment shown in Figure 2J. Significance was quantified by one-way ANOVA. . P values are shown as asterisk: * P ≤ 0.05, ** P ≤ 0.01, *** P ≤ 0.001, **** P ≤ 0.0001.

Supplemental Figure S8: Oxidized mitochondria DNA IL-1β activation is time and TLR9 receptor dependent.

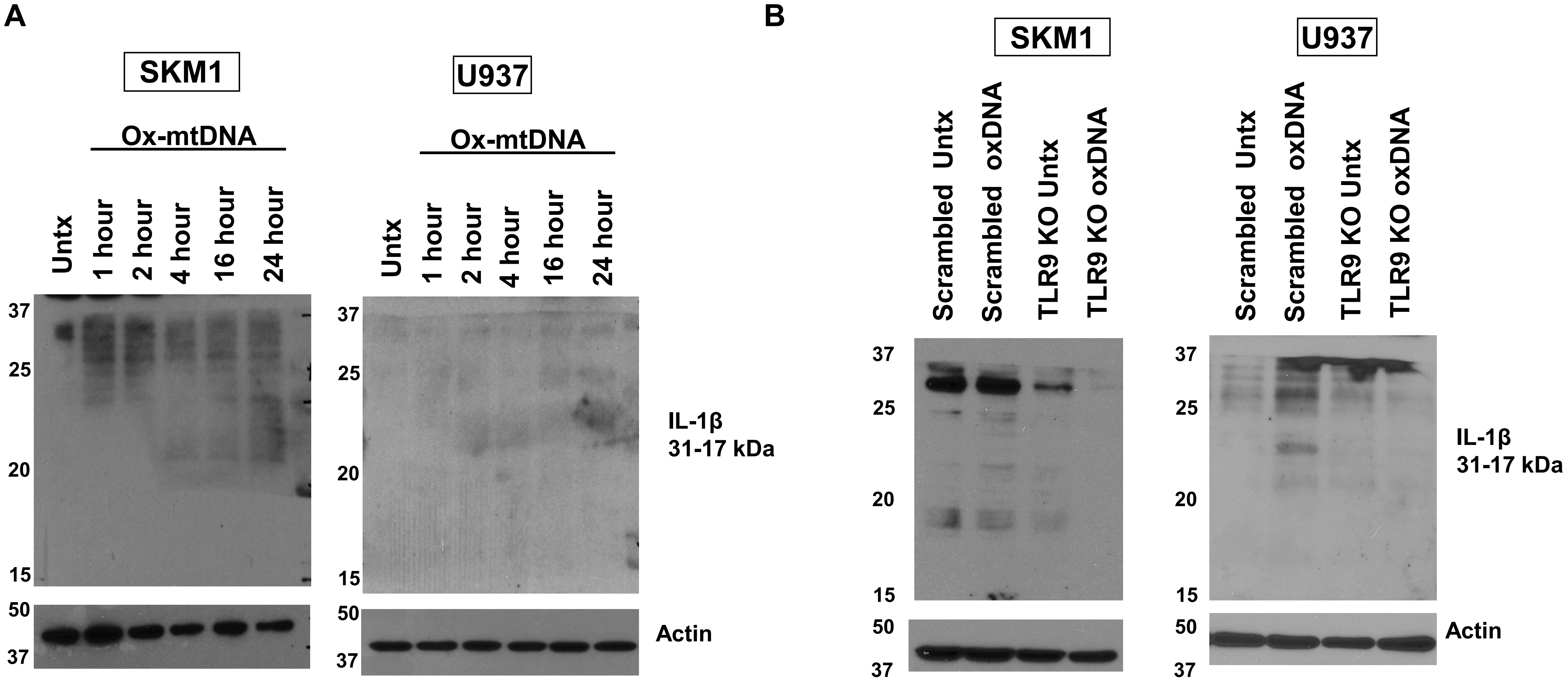

A) Experiment as in Figure 1B testing different doses of ox-mtDNA (as shown) on SKM1 (A) or U937 cells (B) followed by assessment of IL-1β activation (cleavage at 17kDa). LAN (LPS + ATP + Nigericin) was used as a positive control. Figures are representative of n=3 experimental repeats.

Supplemental Figure S9: TLR9 expression in lentiviral transduced cell lines and corroboration of Figure 3 experiments

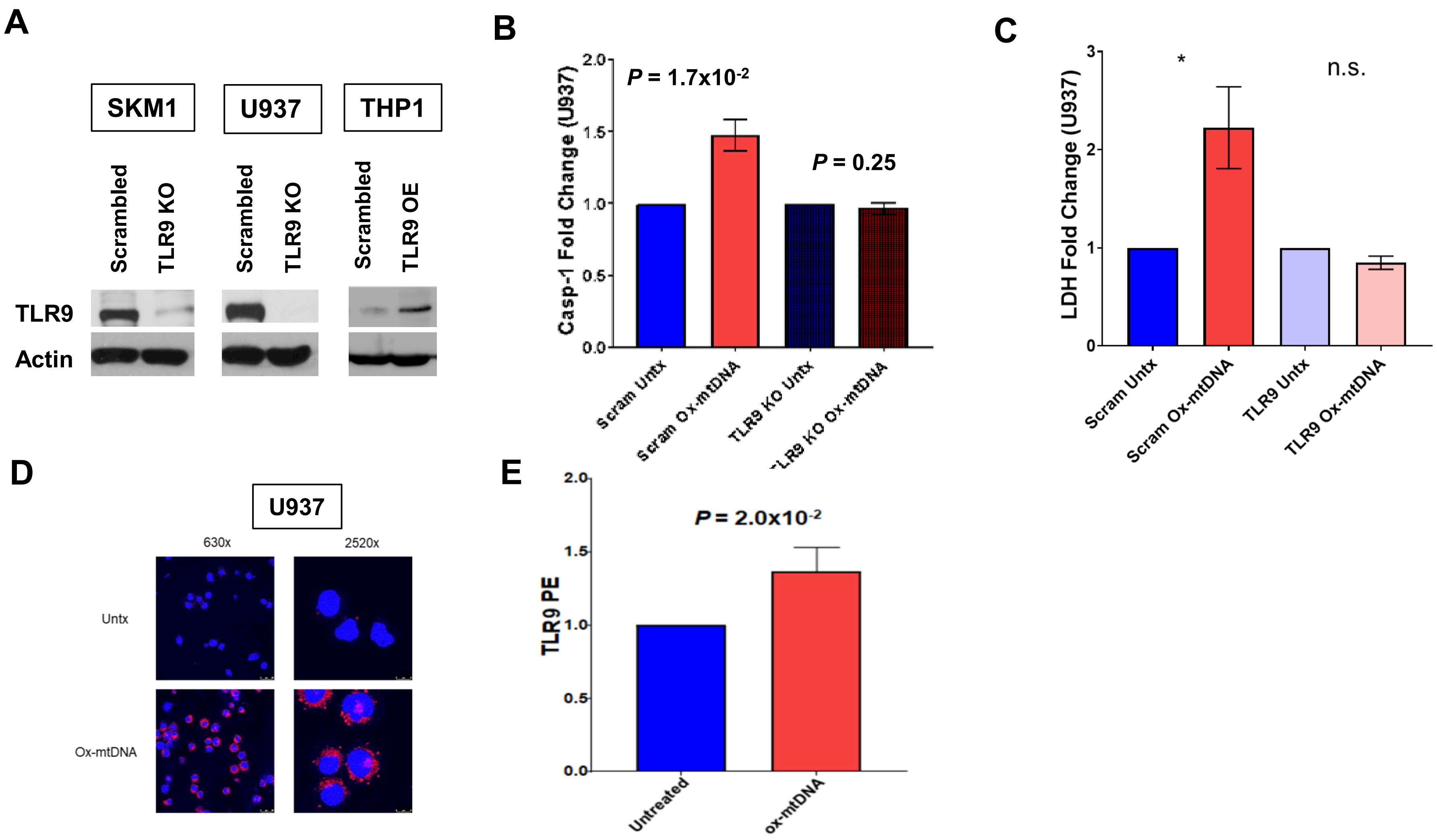

A) Western blot of SKM1 and U937 cells transfected with a lentivirus vector containing TLR9 CRISPR KO guide or western blot of THP-1 cells transfected with a lentiviral vector for TLR9 overexpression. **B)** Experiment as in Figure 3F measuring caspase 1 activation in U937 cells transfected with scrambled or TLR9KO lentivirus. **C)** Experiment as in Figure 3G measuring LDH for proliferation of U937 cells transfected with scrambled or TLR9KO lentivirus. **D)** Experiment as in Figure 3J in U937 cells following lysosomal activation with Lysotracker red after treatment with ox-mtDNA. **E)** Experiment as in Figure 3K in U937 cells showing translocation of TLR9 to the surface of cells after treatment with ox-mtDNA.

Supplemental Figure S10: Nuclear localization of IRF7 after TLR9/ox-mtDNA ligation

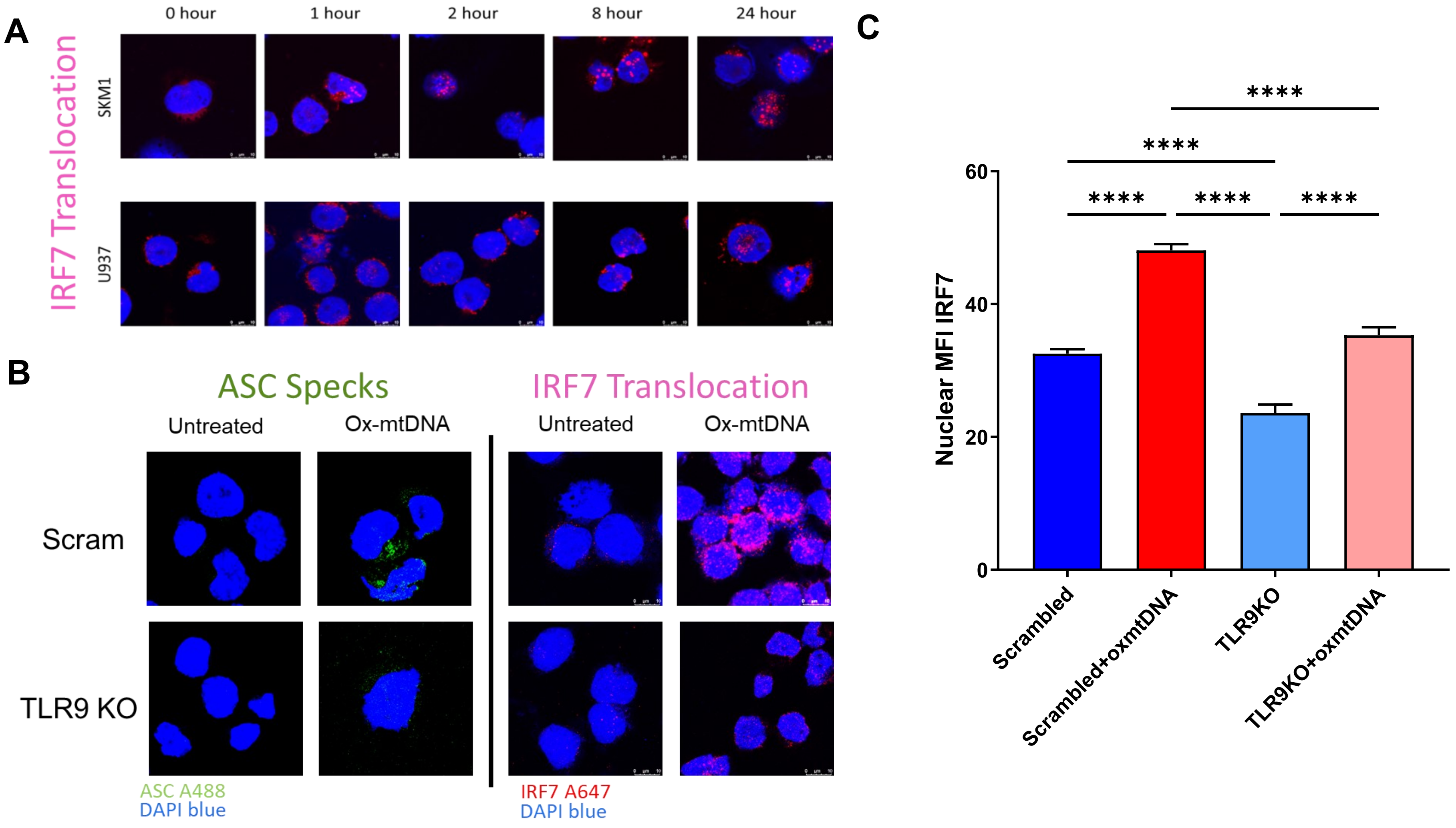

D

NAME	SCORE
CTSB	0.11140112
MAP1LC3C	0.041337512
IRF7	0.038532738
MAP1LC3B	0.001482483
IRF3	-0.038418442
MAP1LC3A	-0.094965264
TBK1	-0.13736689
MAP1LC3B2	-0.5967795

A) Immunofluorescence time lapse of IRF7 nuclear localization in either SKM1 or U937 cells after treatment with ox-mtDNA. **B)** Experiment as in Figure 4 D and E showing immunofluorescence staining of U937 cells transfected with a lentivirus vector containing TLR9 CRISPR KO guide, or a scrambled control) for the formation of ASC specks (green) or IRF7 (red). **C)** Quantification of nuclear IRF7 MFI from pictures in experiment in Figure 4F. **D)**Analysis of previously published RNA-seq data comparing normalized gene read counts from healthy versus MDS BM-MNC showing genes linked to TLR9 activation including IRF7.

Supplemental Figure S11: ISG expression is active in MDS

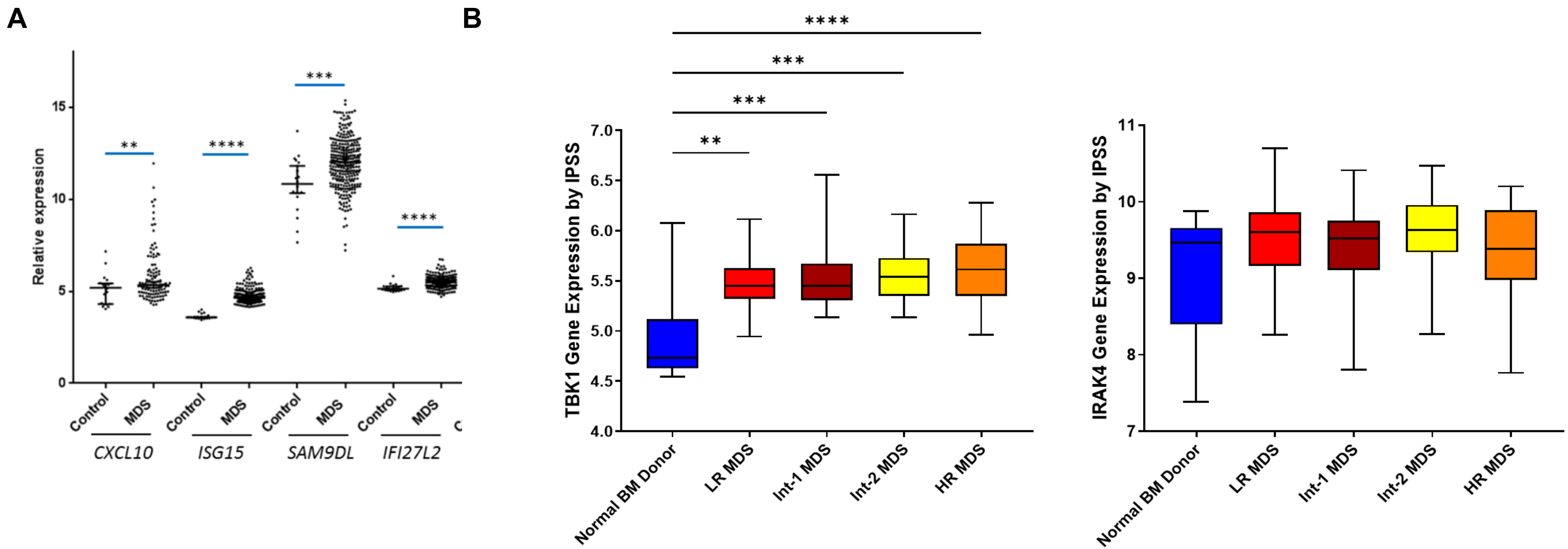

Additional ISG, IFN, and relevant protein assessed by gene expression array (MDS N=213, BMT donors N=20). **(A)** Gene expression array of interferon stimulated genes: CXCL10, ISG15, SAM9DL and IFI27L2 **(**MDS N=213, BMT donors N=20) **(B)** TBK1 and Interleukin 1 Receptor Associated Kinase (IRAK)4 gene expression **(**N=213, BMT donors N=20).

Supplemental Figure S12: U937 ISG activation in response to ox-mtDNA

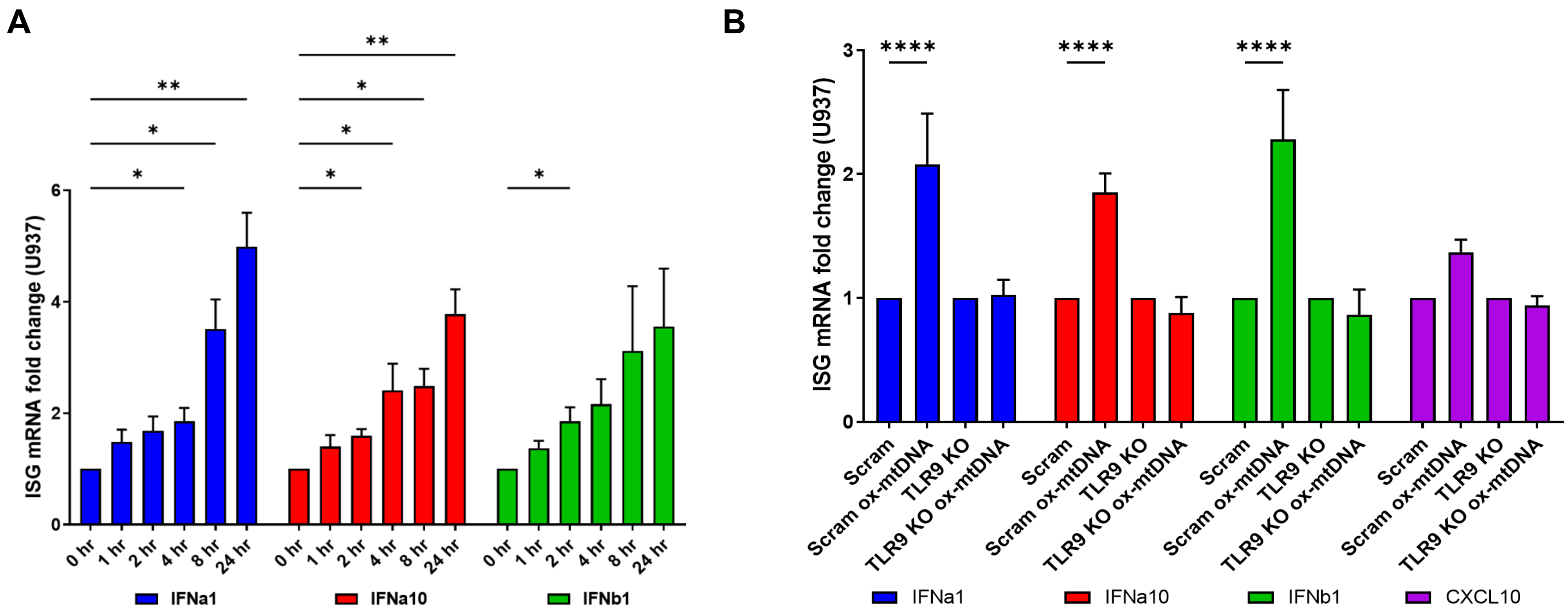

A) Experiment as in Figure 4 H and I showing type I IFN gene expression responses after treatment with 50ng/mL ox-mtDNA for 1-24 hours in U937 cells (Mean fold change ± SD, N=3). **B)** Following ox-mtDNA treatment, IFNa1, IFNa10, IFNb1, and the ISG CXCL10 are all increased by qPCR in normal TLR9 expression but the the fold change in mRNA was lost with TLR9 KO.

Supplemental Figure S13: ox-mtDNA/TLR9 a targetable axis in primary MDS specimens ex vivo

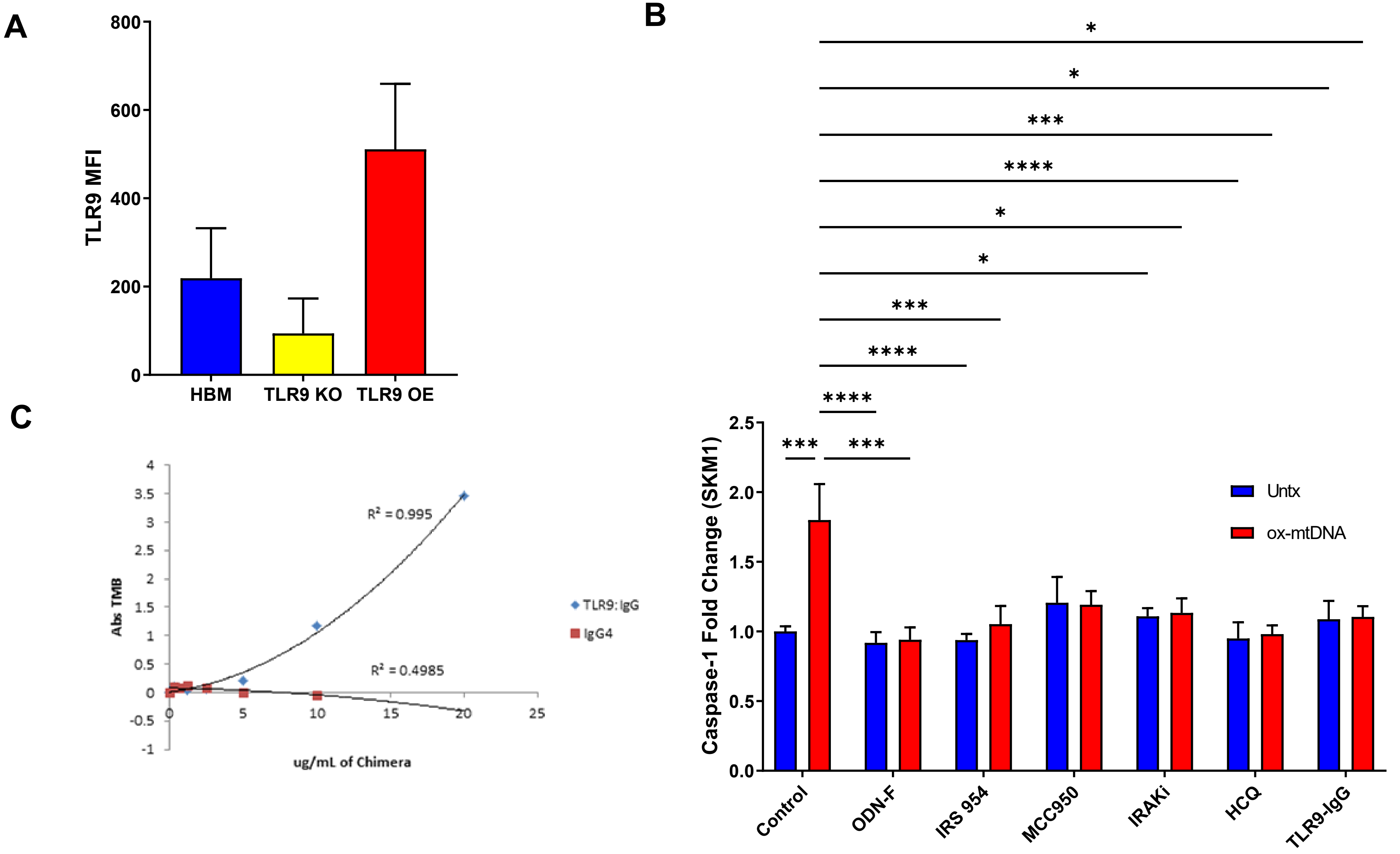

A) Flow cytometric TLR9 expression analysis after transfection in primary healthy BM-MNC transfected with lentiviral vectors. **B)** Fold change of caspase-1 activity of SKM1 cells pre-treated with ox-mtDNA and TLR9 inhibitors (IRS 954, HCQ, ODNF, TLR9-IgG), MCC950, an IRAK-inhibitor to indirectly block inflammasome activation (IRAKi), and controls (Vehicle, IgG). (mean ± SEM of n=3). **C)** Binding capacity of TLR9-igG chimera.
